# A novel metric of self and informant reported cognitive complaints in subjective cognitive decline and mild cognitive impairment

**DOI:** 10.1002/alz70857_107053

**Published:** 2025-12-25

**Authors:** Kristoffer Romero, Ana Badal, Astrid Coleman, Renée K Biss

**Affiliations:** ^1^ University of Windsor, Windsor, ON, Canada; ^2^ York University, North York, ON, Canada

## Abstract

**Background:**

Given the role of subjective cognitive complaints (SCCs) in diagnosing mild cognitive impairment (MCI), there is interest in determining which cognitive complaints best differentiate between older adults with MCI versus those with age‐normal cognition (i.e., with complaints reflecting subjective cognitive decline [SCD]). Rather than analyzing questionnaire‐based SCC data, we examined patient and informant cognitive complaints from open‐ended questions during clinical interview. By conducting multivariate analysis using the verbatim responses provided by patients and their families, we sought to reveal new insights into which presenting SCCs best predict clinical group membership and correlate with objective cognitive performance.

**Method:**

A chart review was conducted on 168 patients meeting diagnostic criteria for MCI (*n* = 91) or research criteria for SCD (*n* = 77) from a tertiary memory clinic in Toronto, Canada. From each report, we coded the presence or absence of SCCs grouped by cognitive domain (e.g., memory, word‐finding) or mood (e.g., depression, anxiety). We then analyzed SCCs of patients and informants separately using multiple correspondence analysis, which derives latent factors that represent which cognitive complaints tend to be reported together. We also explored whether these factors predicted clinical group membership, and were correlated with demographic variables and neuropsychological test scores.

**Result:**

For informant SCCs, the analysis yielded two factors explaining 47% of the variance. The primary factor consisted of cognitive complaints regarding episodic memory, prospective memory, orientation, and navigation; this factor significantly differed between SCD and MCI groups and correlated negatively with delayed recall scores. For self‐reported SCCs, two factors explained 30% of the variance, with the first factor consisting of word‐finding, attention, memory, and depression complaints. Interestingly, this factor was negatively correlated with age and positively correlated with delayed recall.

**Conclusion:**

Using a novel method of quantifying SCCs reported during clinical interview, we found informant concerns regarding memory, orientation, and navigation differentiated between SCD and MCI. Self‐reported SCCs reflected depressive symptoms and broad cognitive concerns, but nonetheless also correlated with objective performance. Informant‐based SCCs may have more utility for diagnosis, but self‐report SCCs can reflect objective performance. A combination of approaches to quantifying SCCs clinically may better characterize cognitive decline in older adults.

